# Immediate implant placement in Type II socket using vestibular socket therapy with pericardial membrane versus simultaneous guided bone regeneration (randomized controlled clinical trial)

**DOI:** 10.1186/s12903-026-08626-7

**Published:** 2026-05-26

**Authors:** Marwan M. Ebrahim, Ahmed M. Shabaan, Yasmine Y. Gaweesh, Esraa A. ElMekkawy, Aya A. Sakr

**Affiliations:** 1 Faculty of Dentistry, Pharos University, Alexandria, Egypt; 2 Department of Oral and Maxillofacial Surgery, Faculty of Dentistry, Alexandria University, Alexandria, Egypt; 3 Department of Periodontology, Faculty of Dentistry, Alexandria University, Alexandria, Egypt; 4 Department of Oral Radiology, Faculty of Dentistry, Alexandria University, Alexandria, Egypt; 5 Maxillofacial Surgery Department, Faculty of Dentistry, Alexandria University, Champollion St., Azarita, Alexandria, 21527 Egypt

**Keywords:** Immediate implant, Type-II socket, VST, GBR

## Abstract

**Introduction:**

Immediate implant placement with flap elevation and guided bone regeneration in Type II sockets often leads to midfacial gingival recession and compromised esthetics. To address these limitations, vestibular socket therapy (VST) has been proposed as an alternative technique. Our objective is to compare vestibular socket therapy using pericardium membrane versus conventional flap and guided bone regeneration (GBR) for immediate implant placement in type II sockets regarding bone and soft-tissue outcomes.

**Materials and methods:**

Twenty patients with Type II sockets were divided into two equal groups. Group-I: received immediate implant placement through a vestibular incision with pericardium membrane placement; the defect was grafted with a mixture of allograft and xenograft. Group-II: received immediate implant placement with conventional open-flap surgery using the same membrane and grafting materials. Radiographic evaluation of labial bone was performed at 3 and 6 months using CBCT assessments. Volumetric analyses of buccal soft-tissue contours were recorded preoperatively and at 6 months. Clinical assessment of pink esthetic score (PES) and modified sulcus bleeding index (mSBI) also were performed at 6 months.

**Results:**

Both groups showed statistically significant increases in buccal bone thickness and height from baseline to 3 and 6 months. Volumetric change and mSBI values demonstrated no statistically significant differences between groups (*p* > 0.05). Mean PES was significantly higher in the study group (12.78 ± 0.83) compared to controls (11.56 ± 1.24) (*p* = 0.03).

**Conclusions:**

Pericardium membrane with VST offers a promising approach for immediate implant placement in type-II sockets, achieving predictable bone regeneration and better esthetic outcomes, as reflected by higher PES.

**Trial registration:**

Retrospectively registered at ClinicalTrials.gov (NCT07337837) (13/1/2026).

## Introduction

Tooth replacement using immediate implants has become a frequently used clinical procedure [1, 2]. For successful immediate implant placement with high survival rates, ideal anatomical conditions are required. The labial bone should be thick and intact, and a thick gingival phenotype is preferable [3].

Several studies have proposed techniques to achieve predictable esthetic outcomes for immediate implant placement in Type II sockets, as classified by Elian et al. [4]. The features of a Type II socket are intact facial soft tissue despite partial or complete loss of the facial bone plate. Immediate implant placement with GBR is one approach that has demonstrated favorable outcomes; however, the need for extensive flap reflection and significant flap advancement to address soft-tissue deficiencies is a notable drawback. Additionally, Liu et al. reported that this approach may lead to midfacial recession and compromised soft-tissue esthetics [5]. 

“Vestibular socket therapy” [6] is a recently emerging technique that enables immediate implant placement in Type II sockets. A horizontal vestibular access incision is created 4 mm apical to the mucogingival junction instead of the open-flap surgery inherent to GBR. A mucoperiosteal tunnel is then elevated, extending from the socket orifice into which a cortical slowly resorbabing bone laminar allograft is inserted and secured beneath the tunnel. This configuration creates a stable, graft-able. jumping gap between the implant surface and the future reconstructed labial plate, promoting adequate labial bone thickness upon complete healing [7].

The use of cortical bone lamina has several drawbacks. Its limited flexibility requires precise trimming and contouring, which can prolong surgery and increase placement errors [8, 9]. Additionally, its slow resorption rate makes it more susceptible to exposure when soft tissue coverage is inadequate due to inadequate flap advancement [10, 11]. Higher costs may also limit its use as compared to more affordable resorbable collagen membranes [12]. Although no serious complications have been reported in VST applications, exploring alternatives that address these limitations remains necessary.

Among the available resorbable barrier materials, pericardium-derived collagen membranes provide several clinical advantages over cortical laminae. It has great elasticity, enhanced adaptability to complex defects, and reduced exposure risk [13, 14].

Based on the documented advantages of pericardial membranes and disadvantages of bone cortical laminae, a modification of the VST was explored in this study with the purpose of evaluating labial bone augmentation following immediate implant placement in Type II sockets using a pericardium collagen membrane (study group) compared to GBR with open-flap surgery (control group). Our null hypothesis was that no significant differences would occur in labial bone thickness between the two modalities.

## Materials and methods

### Study population

This study was conducted as a randomized controlled clinical trial at the Oral and Maxillofacial Surgery Department, Faculty of Dentistry, Alexandria University, Egypt. Adult participants were recruited from the department’s outpatient clinic if they were wishing to have immediate implant placement of a single hopeless non-molar maxillary tooth.

### Inclusion and exclusion criteria

Patients were those with single, non-restorable maxillary non-molar teeth with Type II sockets confirmed by Cone Beam Computed Tomography (CBCT). Exclusion criteria included heavy smoking (over 10 cigarettes daily), the presence of acute periapical infection, poor oral hygiene, pregnancy, or any systemic condition that could compromise surgical outcomes or healing.

#### Ethical approval

Ethical approval for this study was obtained from the Ethics Research Committee of the Faculty of Dentistry, Alexandria University (IRB 00010556 – IORG 0008839; approval number 0809 − 12/2023). The study was conducted in accordance with the ethical principles of the Declaration of Helsinki version 2024. Participants were adequately informed about all study-related procedures, and written informed consent was signed prior to surgery. The data are reported in accordance with the Consolidated Standards of Reporting Trials (CONSORT) statement for standardized reporting of randomized clinical trials. The trial was retrospectively registered at ClinicalTrials.gov (NCT07337837) (13/1/2026).

### Sample size estimation

Sample size was calculated based on a 95% confidence level, 5% alpha error, and 80% study power to detect differences in buccal plate bone thickness following immediate implant placement. The primary endpoint for sample size estimation was the difference in buccal plate bone thickness at the at 6 months postoperatively. Elaskary et al. [6] reported a mean (SD) buccal bone thickness of 2.34 mm (0.78) at 6 months following vestibular socket therapy in patients with deficient facial plates. In addition, Liu et al. [5] reported mean (SD) buccal bone thickness values of 3.25 mm (1.37) immediately postoperatively and 2.31 mm (1.13) at 12 months, corresponding to a mean reduction of 0.94 mm (SD 0.51) at the coronal level, measured using CBCT. Based on comparison of means, and using the highest reported standard deviation to ensure a conservative and adequately powered estimate, the minimum required sample size was calculated to be 8 patients per group, increased to 9 patients per group to compensate for anticipated loss to follow-up. The total required sample size= number of groups × number per group = 2 × 9 = 18 patients [15]. MedCalc Statistical Software version 19.0.5 (MedCalc Software bvba, Ostend, Belgium) was used.

### Grouping

**Fig. 1:**
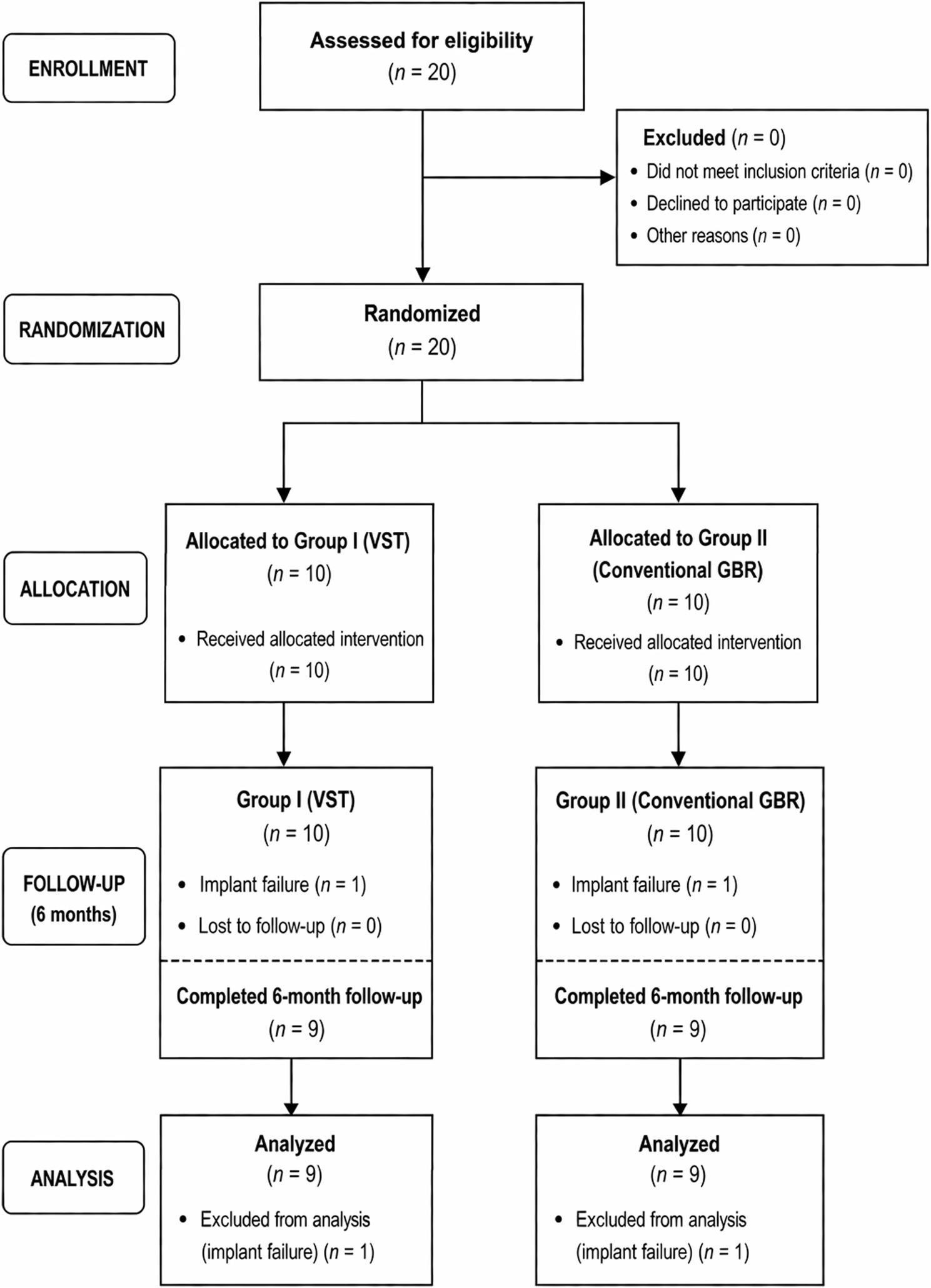
Consort flow diagram of patient recruitment, allocation, follow-up, and analysis

Following patient enrollment, they were divided into two groups (Fig. 1).

- Group I: received immediate implant placement through a modified VST.
- Group II: received immediate implant placement with conventional open-flap surgery to facilitate guided bone regeneration.

Random sequence generation was performed using simple randomization through computer-generated random numbers by an investigator (E.A.E) who was not involved in patient recruitment or surgical procedures. Allocation concealment was ensured using sequentially numbered, opaque, sealed envelopes prepared by the same independent investigator. These envelopes were delivered to the operating surgeon and were opened only at the time of surgery immediately before the intervention.

After enrollment, eligible participants who provided informed consent were randomly assigned in a 1:1 allocation ratio between the groups according to the generated sequence.

Due to the inherent differences between the surgical techniques, blinding of the operating surgeon was not feasible. However, participants, the outcome assessor responsible for clinical and radiographic measurements, and the statistician performing the data analysis were blinded to the group allocations.

### Clinical and radiographic procedures

#### Preoperative assessment

**Fig. 2:**
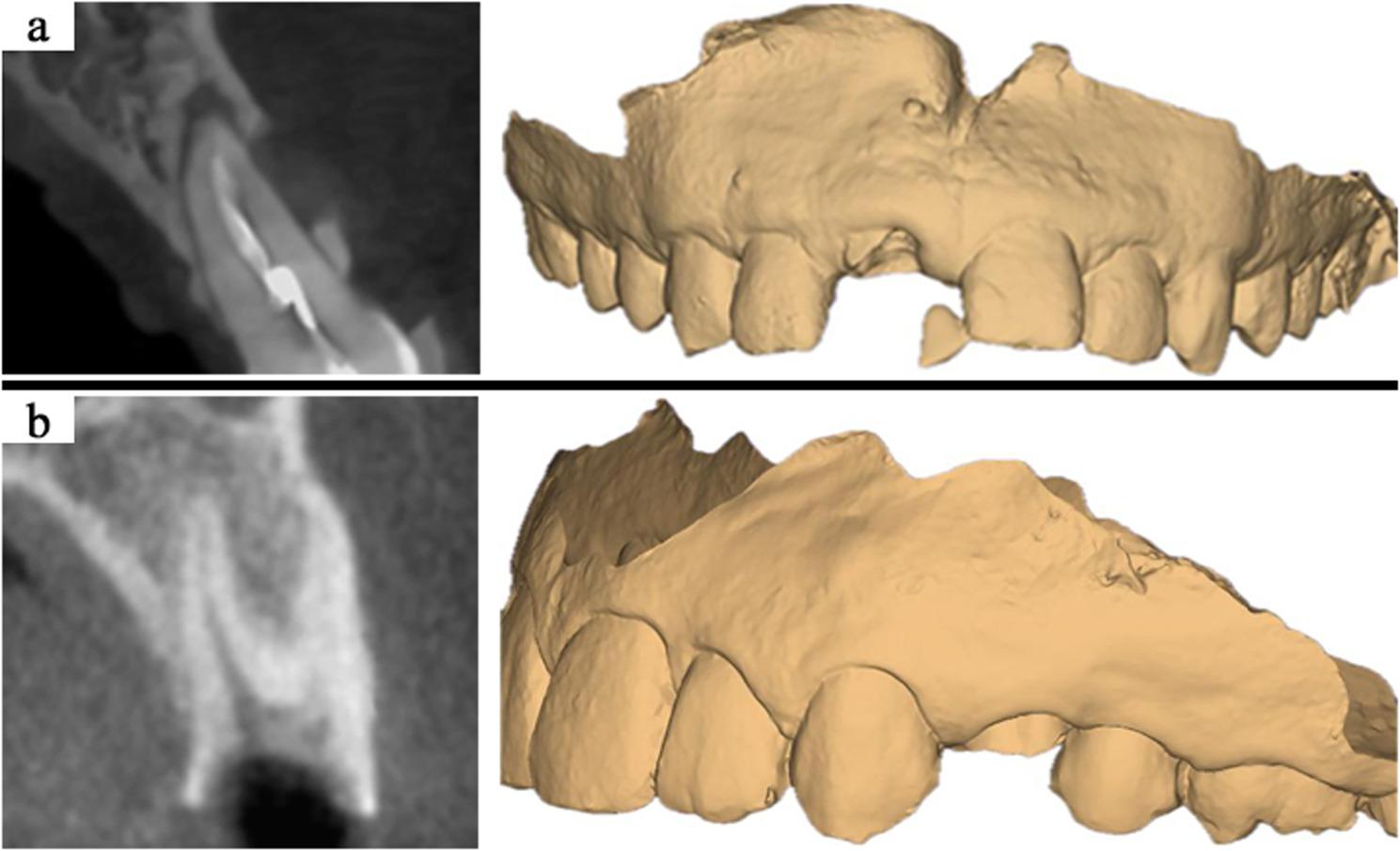
**A** Pre-operative CBCT and intra-oral scans for study group. **B** Pre-operative CBCT and intra-oral scans for control group

Preoperative CBCT was performed for implant surgical planning and to measure labial bone heights and thicknesses. Buccal soft-tissue contours were recorded preoperatively using an intraoral scanner (Omnicam, Dentsply Sirona, USA) for volumetric analysis during the follow-up phase (Fig. 2).

#### Surgical procedures

For both groups, atraumatic tooth extraction was carried out under local infiltration anesthesia. Thorough socket curettage was performed.

**Fig. 3:**
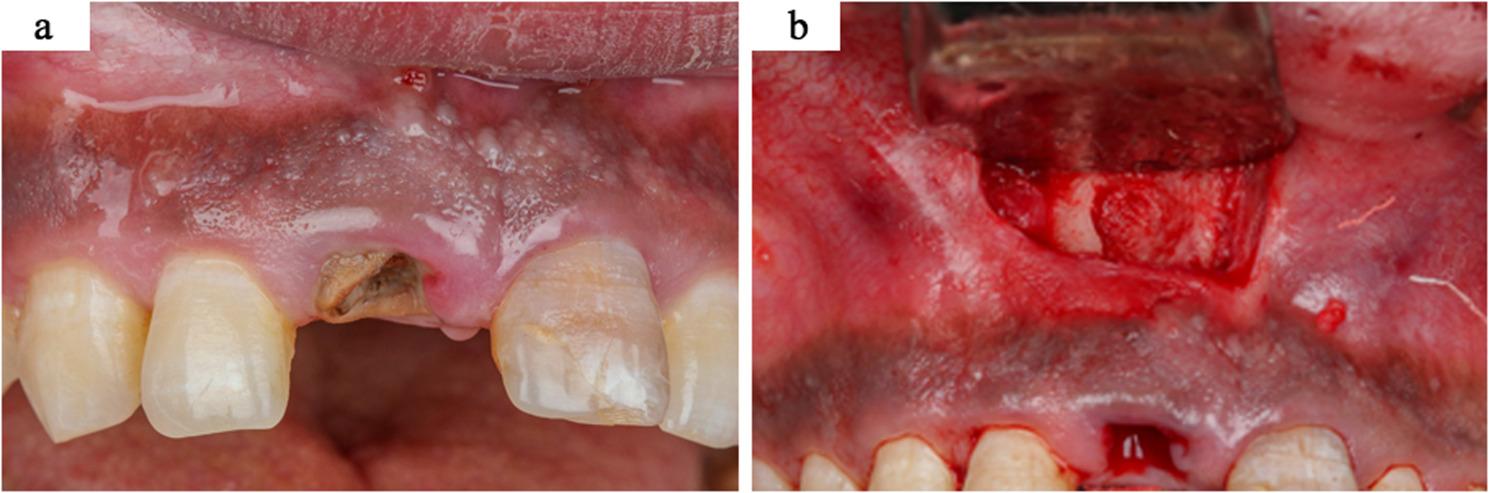
**A** Hopeless maxillary right central incisor. **B** Atraumatic extraction and vestibular access incision

For the modified VST group, a horizontal incision was created in the vestibule, approximately 5–10 mm long and positioned 3–4 mm apical to the mucogingival junction. A sub-mucoperiosteal tunnel then was made, beginning at the labial aspect of the gingival margin and extending apically to connect with the vestibular incision (Fig. 3).

**Fig. 4:**
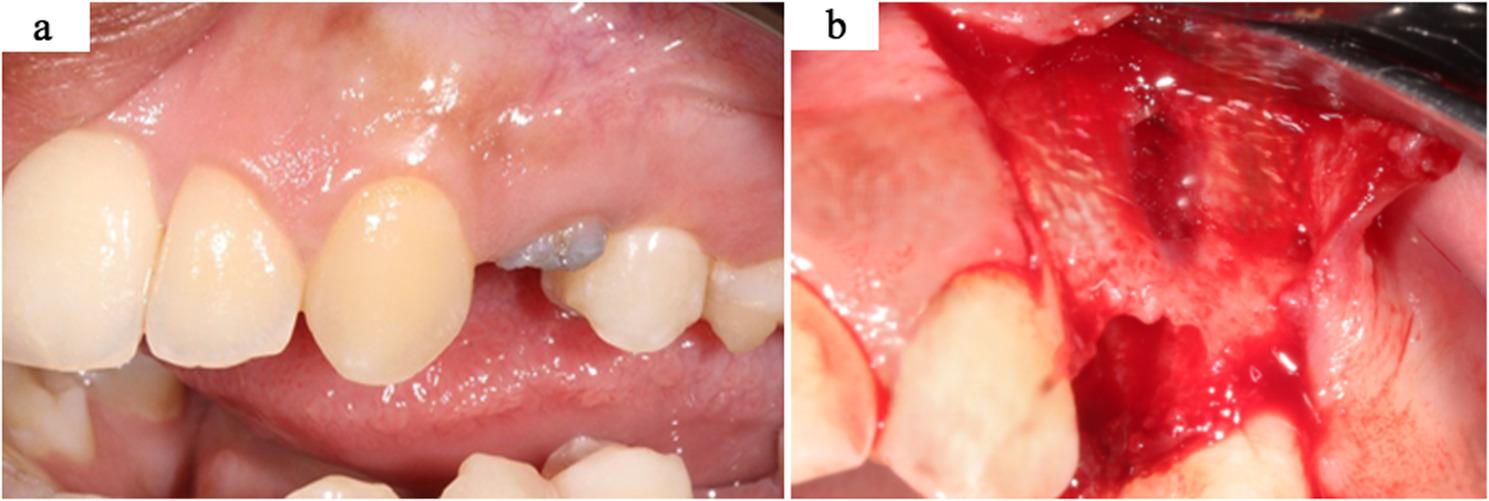
**A** Hopeless maxillary left first premolar. **B** Atraumatic extraction and a facial vertical releasing incision was made at the distal aspect of the adjacent tooth

For the control group, a labial marginal releasing incision was made at the distal aspect of the neighboring tooth, extending to just below the mucogingival junction to allow elevation of a full-thickness triangular mucoperiosteal flap (Fig. 4).

**Fig. 5:**
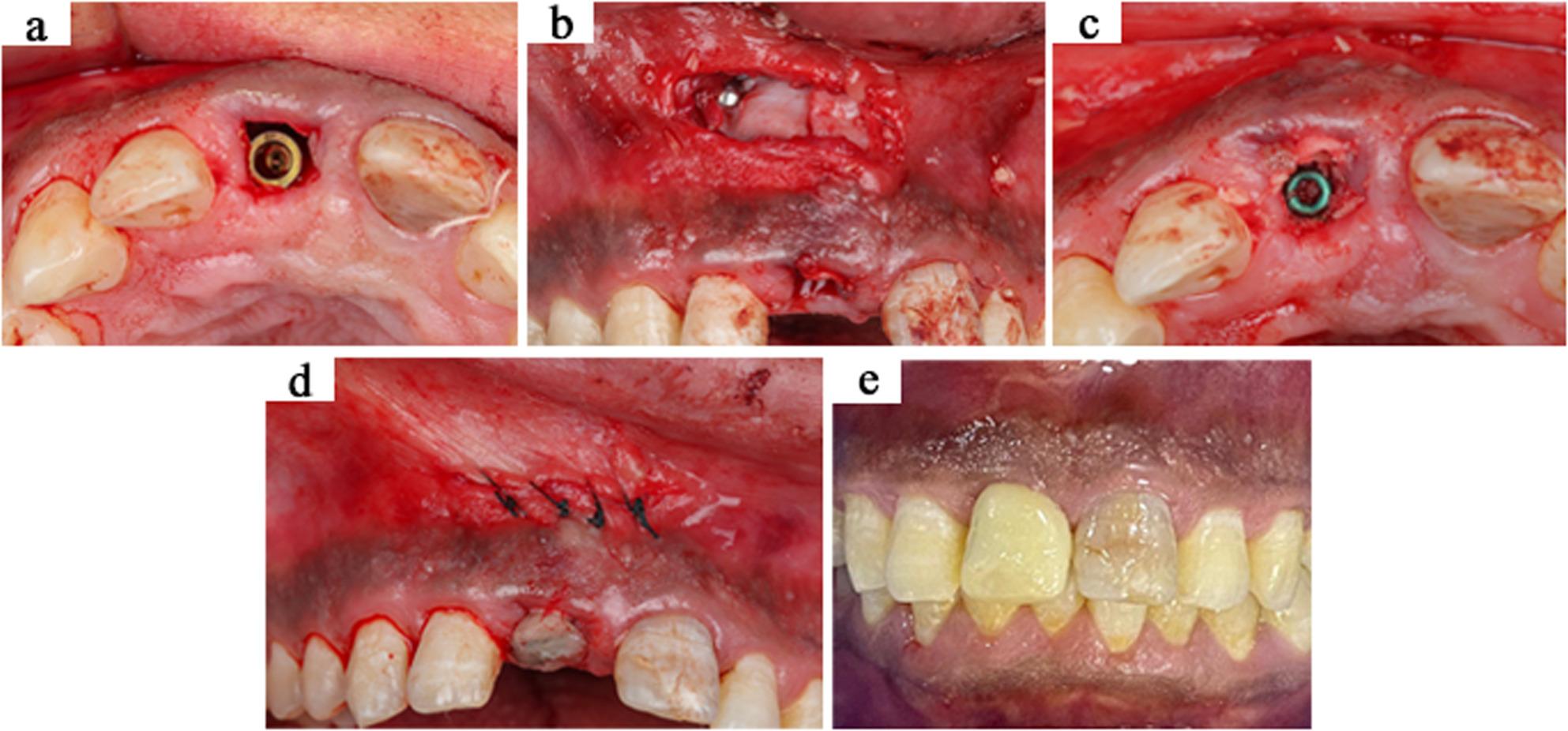
**A** Implant placed with jumping gap. **B** Pericardium membrane inserted and fixed with bone tacks. **C** Bone graft packed in jumping gap between the implant and the membrane. **D** 4/0 silk sutures used for vestibular incision closure and anatomical healing abutment used for socket sealing. **E** Definitive restoration

**Fig. 6:**
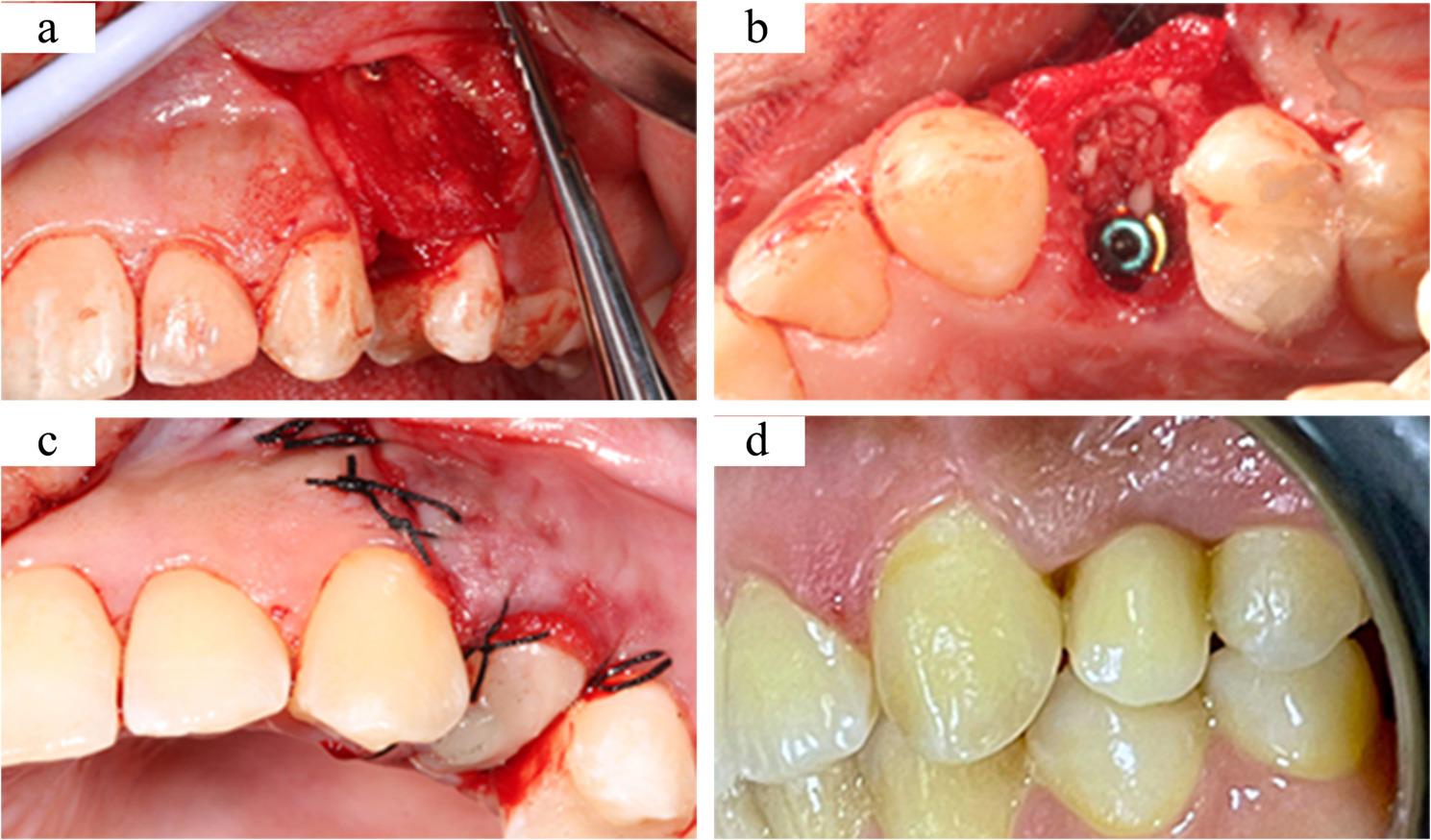
**A** Pericardium membrane inserted and fixed with bone tacs. **B** Implant placed and jumping gap packed with bone graft. **C** 4/0 silk sutures used for flap closure and anatomical healing abutment used for socket sealing. **D** Definitive restoration

Osteotomy was performed for both groups following the manufacturer’s protocol for the selected implant system (ROOTT, Switzerland). The implant was placed palatally, 4 mm apical to the labial gingival margin with engagement of 3.0 to 4.0 mm of apical bone to achieve initial stability. A 0.4 mm thick pericardium membrane (Shelter F UBGEN, Italy) was trimmed to cover the defect and extend beyond 2 mm from its margins, inserted, and secured in place using membrane tacks (G-Tacks, Garantie, Germany). Labial gaps were packed with a combination of allograft materials (Regain-Oss Allograft, Garantie, Germany) and xenograft bone materials (Regain-Oss Xenograft, Garantie, Germany).The horizontal incision in each site was closed using 4 − 0 silk sutures after customized composite healing abutments were inserted to achieve adequate socket orifice sealing. Prior to insertion, these abutments were appropriately contoured and polished to support optimal soft-tissue emergence (Figs. 5 and 6).

### Postoperative management

Patients were advised to apply ice packs during the first day. They were also recommended to rinse twice daily with 0.2% chlorhexidine gluconate mouth rinse for 5 days. For 7 days, patients were given amoxicillin–clavulanic acid (1 g every 12 h). Additionally, an anti-edematous enzyme combination (Trypsin 300 E.A.U. and Chymotrypsin 300 E.A.U. every 8 h for 3 days) and a nonsteroidal anti-inflammatory drug (ibuprofen 600 mg every 8 h for 3 days) were provided. Sutures were removed after 7 days.

### Outcome measures and measurement standards

#### Buccal bone thickness and height

**Fig. 7:**
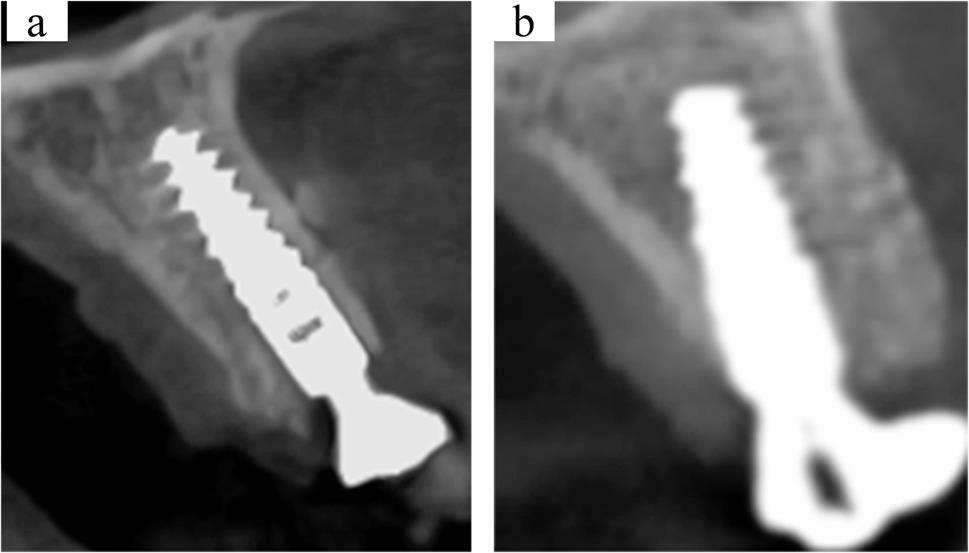
**A** Study group: 3 months CBCT scan, (**B**) Control group: 3 months CBCT scan

At 3- and 6-months following surgery, CBCT scans were taken to assess changes in buccal bone height and thickness (Fig. 7). Buccal bone thickness was measured as the distance from the implant outer surface to the outer limit of cortical bone on these 3- and 6-month scans, and as the distance from root surface to the outer labial cortical bone at baseline CBCT scan. Thicknesses were measured at three levels: crestal level, 3 mm apical, and 6 mm apical to the crestal level. Labial bone height was measured from the apical end of the implant (projected on baseline) to the labial bone crest [7].

#### Volumetric analysis

Intraoral scans were repeated 6 months postoperatively in order to create digital STL surface models. Pre- and post-operative STL files were imported into Cloud Compare (version 2.6.2; open-source software) for spatial alignment. Non-surgerized teeth were used as anatomical landmarks for initial point-pair registration, followed by fine adjustment until a root mean square error < 0.5 was achieved. The aligned meshes were then exported to Meshmixer (Autodesk Inc., USA) for solidification to generate watertight models suitable for volumetric analysis. In 3D Matic (version 13, Materialise NV, Belgium), a standardized region of interest was defined using reproducible anatomical landmarks, and both models were cropped accordingly. Final volumes were calculated in Matic for quantitative assessment and statistical comparison [16].

#### Pink esthetic score

At the 6-month follow-up visit, PES was recorded to evaluate mesial papilla, distal papilla, soft-tissue color, texture, level, contour, and alveolar process deficiency [17].

#### Modified sulcus bleeding index

At 6-months, each buccal and palatal surface received a score from 0 to 3, and the mean was recorded. The score was recorded according to the following criteria [18]:

- Score 0: No bleeding upon passage of a periodontal probe along the gingival margin surrounding the implant.
- Score 1: Isolated bleeding spots visible.
- Score 2: Blood forms a red line on the margin.
- Score 3: Heavy bleeding.
- All radiographic, volumetric were performed by a single experienced examiner. To assess intra-examiner reliability, measurements were repeated after a two-week interval, and the intraclass correlation coefficient was calculated. The values ranged from 0.898 to 0.992, indicating excellent reliability [19].

### Statistical analysis

Descriptive statistics were reported as means and SD and as medians with interquartile ranges (IQR). Data normality was evaluated using descriptive measures, Q–Q plots, histograms, and the Shapiro–Wilk test. Between-group differences were assessed using the independent samples t-test for normally distributed variables (volumetric change and total pink esthetic score) and the Mann–Whitney U test for non-normally distributed variables (labial bone thickness and height, bleeding index, and pink esthetic score subscales). Within-group comparisons across different time intervals were analyzed using the Friedman test, followed by Bonferroni-adjusted pairwise comparisons. A significance level of *p* < 0.05 was adopted. All statistical analyses were conducted using IBM SPSS Statistics for Windows, Version 26.0 (IBM Corp., Armonk, NY, USA).

## Results

20 patients met the inclusion criteria: 8 males and 12 females, aged 18 to 53 years old. A total of 20 implants were placed, distributed as follows: 9 in maxillary central incisors (5 in Group I and 4 in Group II), 2 in maxillary lateral incisors (1 in each group), 2 in maxillary canines (1 in each group), and 7 in maxillary premolars (3 in Group I and 4 in Group II).Two implants failed to osseointegrate during the healing phase, with one failure occurring in each group; these cases were excluded from the 6 months analysis No other post-operative complications such as infection or membrane exposure, were detected in either group.

### Buccal bone thicknesses

Both groups had a statistically significant increase in buccal bone thickness from baseline to 3 and 6 months across all measured levels, with no significant differences between the 3- and 6-month evaluations (*P* > 0.05). An exception was noted in the study group at the 0-mm level, where no significant change was observed over time. The pattern of bone gain was comparable in both groups, with no statistically significant differences at any follow-up points (Table 1).

**Table 1:** Comparison of bucal bone thicknesses

			Test	Control	*P* value 1
0 mm	0 months	Mean (SD)	0.40 (± 0.49)	0.36 (± 0.45)	0.86
		Median (IQR)	0.00 (0.00, 0.90)	0.00 (0.00, 0.85) a	
	3 months	Mean (SD)	1.03 (± 0.72)	1.79 (± 1.19)	0.19
		Median (IQR)	0.80 (0.75, 1.30)	1.00 (0.80, 3.00) b	
	6 months	Mean (SD)	0.91 (± 0.56)	1.56 (± 1.43)	0.39
		Median (IQR)	0.80 (0.65, 1.25)	1.00 (0.00, 3.05) ab	
3 mm	0 months	Mean (SD)	0.40 (± 0.32)	0.83 (± 0.64)	0.10
		Median (IQR)	0.50 (0.00, 0.65) a	0.80 (0.25, 1.30) a	
	3 months	Mean (SD)	1.30 (± 0.44)	2.03 (± 1.16)	0.34
		Median (IQR)	1.10 (1.00, 1.50) b	1.20 (1.00, 3.25) b	
	6 months	Mean (SD)	1.28 (± 0.36)	2.06 (± 1.19)	0.44
		Median (IQR)	1.20 (1.00, 1.50) b	1.30 (1.00, 3.35) b	
6 mm	0 months	Mean (SD)	0.43 (± 0.35)	0.63 (± 0.45)	0.34
		Median (IQR)	0.60 (0.00, 0.75) a	0.70 (0.25, 0.80) a	
	3 months	Mean (SD)	1.68 (± 0.65)	1.90 (± 0.99)	0.80
		Median (IQR)	1.50 (1.35, 2.05) b	1.30 (1.10, 2.70) b	
	6 months	Mean (SD)	1.76 (± 0.78)	1.88 (± 1.07)	0.80
		Median (IQR)	1.50 (1.35, 2.25) b	1.30 (1.00, 3.00) b	

*SD* Standard Deviation, *IQR *Interquartile range

*P* value 1: Comparison between the test and control using Mann-Whitney U test

a-b: different letters denote significant differences between timepoints using Bonferroni correction

### Buccal bone heights

Buccal bone height showed a statistically significant increase from baseline to 3 and 6 months within each group (*P* < 0.001). No significant difference was observed between the 3- and 6-month measurements (*P* > 0.05). Although baseline values differed significantly between groups (*P* = 0.02), no statistically significant intergroup differences were observed at 3 or 6 months (*P* > 0.05).Regarding the relative change, the mean percentage increase in buccal bone height was higher in the test group (169.23 ± 165.65) compared to the control group (60.08 ± 47.10); however, this difference was not statistical significant (*P* = 0.21) (Table 2).

**Table 2:** Comparison of buccal bone height

		Test	Control	*P* value 1
0 months	Mean (SD)	4.42 (± 3.05)	8.01 (± 2.15)	0.02*
	Median (IQR)	5.60 (1.00, 7.00) a	9.00 (5.30, 9.75) a	
3 months	Mean (SD)	12.03 (± 1.42)	12.96 (± 0.95)	0.16
	Median (IQR)	13.00 (10.30, 13.00) b	13.00 (12.75, 13.65) b	
6 months	Mean (SD)	11.90 (± 1.35)	12.78 (± 1.12)	0.11
	Median (IQR)	12.50 (10.20, 13.00) b	13.00 (12.70, 13.30) b	
Percent change	Mean (SD)	169.23 (165.65)	60.08 (47.10)	0.21
	Median (IQR)	85.71 (72.41, 195.45)	67.37 (36.06, 113.39)	
*P* value 2		< 0.001*	< 0.001*	

*SD* Standard Deviation, *IQR *Interquartile range

*P* value 1: Comparison between the test and control using Mann-Whitney U test

*P* value 2: Comparison between different timepoints within each group using Friedman test

*Statistically significant at *p*-value <0.05

a-b: different letters denote significant differences between timepoints using Bonferroni correction

### Modified sulcus bleeding index

At 6 months, modified sulcus bleeding index values did not differ significantly between the two groups (*p* > 0.05). The study group demonstrated a mean score of 0.44, compared with 0.78 in the control group.

### Volumetric analysis

Volumetric analysis confirmed preservation of the buccal soft-tissue profile in both groups, with mean volumetric changes of − 21.94 (± 6.21) mm³ for the study group and − 23.83 (± 5.78) mm³ for the control group (*p* > 0.05).

### Pink Esthetic Score (PES)

At 6 months, the study group achieved a significantly higher PES mean = 12.78 (± 0.83) compared to controls = 11.56 (± 1.24) (*p* = 0.03). The superior PES in the study group was primarily attributed to enhanced mesial and distal papillary forms, while the remaining PES components were comparable between groups (Table 3).

**Table 3:** Comparison of pink esthetic score

		Test	Control	*P* value
Pink Esthetic score	Mean (SD)	12.78 (± 0.83)	11.56 (± 1.24)	0.03*
	Median (IQR)	13.00 (12.00, 13.50)	11.00 (10.50, 13.00)	
Mesial papilla	Mean (SD)	2.00 (0.00)	1.44 (± 0.53)	0.01*
	Median (IQR)	2.00 (2.00, 2.00)	1.00 (1.00, 2.00)	
Distal papilla	Mean (SD)	2.00 (0.00)	1.22 (± 0.44)	0.004*
	Median (IQR)	2.00 (2.00, 2.00)	1.00 (1.00, 1.50)	
Soft tissue contour	Mean (SD)	1.78 (± 0.44)	2.00 (± 0.00)	0.44
	Median (IQR)	2.00 (1.50, 2.00)	2.00 (2.00, 2.00)	
Gingival level	Mean (SD)	1.56 (± 0.53)	1.33 (± 0.50)	0.44
	Median (IQR)	2.00 (1.00, 2.00)	1.00 (1.00, 2.00)	
Alveolar process	Mean (SD)	2.00 (0.00)	1.78 (± 0.44)	0.44
	Median (IQR)	2.00 (2.00, 2.00)	2.00 (1.50, 2.00)	
Coloring	Mean (SD)	1.44 (± 0.53)	1.78 (± 0.44)	0.25
	Median (IQR)	1.00 (1.00, 2.00)	2.00 (1.50, 2.00)	
Texture	Mean (SD)	2.00 (0.00)	2.00 (0.00)	1.00
	Median (IQR)	2.00 (2.00, 2.00)	2.00 (2.00, 2.00)	

*SD* Standard Deviation, *IQR *Interquartile range

Mann-Whitney U test was used

*Statistically significant at *p*-value < 0.05

## Discussion

This randomized clinical trial evaluated a modified vestibular socket therapy (VST) approach using pericardium collagen membrane compared with conventional open-flap with guided bone regeneration for managing buccal bone defects following immediate implant placement into to Elian Type II sockets. Both techniques effectively reconstructed the buccal bone plates and supported peri-implant soft-tissue stability, although the modified VST group demonstrated superior PES esthetic outcomes.

The principal difference between the protocol used in the present study and the original vestibular socket therapy (VST) protocol was in the choice of barrier material used. The original VST technique used cortical laminar allograft, while in the present study, a pericardium-derived collagen membrane was selected [6].

Both groups showed significant increases in buccal bone thickness at 3 and 6 months, with no significant differences between the two approaches. The comparable outcomes may be partially attributed to the use of the same grafting materials and barrier membranes in both protocols, despite differences in surgical approach. In the present study, the mean crestal buccal bone thickness after 6 months with the modified VST approach was 0.91 mm which was lower than the 1.72 mm reported by Elaskary et al. after 1 year [7]. This difference may be attributed to the fast resorption rate (4–5 weeks) of pericardium membrane which may not have maintained space long enough to allow the formation of thicker buccal bone, in contrast to the slowly resorbing cortical laminae employed in the original VST technique.

Both groups in the present study also exhibited substantial increases in buccal bone heights during healing, with no significant difference between them. The modified VST achieved a mean buccal bone height gain of 7.5 mm, compared with 6.1 mm reported for the original VST [7]. Although our modified technique produced slightly greater gains, the values were close, suggesting that using different barrier membranes may not have significantly influenced labial bone height outcomes.

Volumetric changes values were similar in both groups in the present study.

Modified sulcus bleeding index scores were relatively low in both groups compared to those of Elaskary et al., using a cortical lamina (mSBI score 1.19) [7].

The most notable difference between the two groups was in esthetic performance. Our modified VST group achieved significantly higher overall PES scores, primarily driven by superior mesial and distal papilla scores, indicating enhanced papillary fill and soft-tissue support. This may relate to the vestibular access and the avoidance of papillary incisions, which are critical for esthetic preservation in the maxillary anterior region. By contrast, no significant differences were found between groups in soft-tissue contour, gingival level, alveolar process, color, or texture, indicating that both techniques produced satisfactory esthetic outcomes overall. The overall Pink Esthetic Scores with our modified VST were comparable to those of the originnal VST, with mean values of 12.78 and 12.63, respectively [7].

Overall, the study group achieved bone regeneration comparable to the conventional open-flap approach while providing superior esthetic outcomes. These findings support VST using pericardium membrane as a predictable, minimally invasive technique for immediate implant placement in the anterior esthetic zone. Long-term investigations are recommended to assess stability beyond an early healing phase of only 6 months.

Despite favorable outcomes, the study has limitations. Longer follow-up is needed to confirm stability, and volumetric analysis may be influenced by intraoral scanning and registration accuracies. The use of a fast-resorbing pericardium membrane may support only limited space maintenance during healing. Future studies should evaluate slower resorbing pericardial membranes to determine their potential impact on labial bone thickness following immediate implant placement. A limitation of this study is the retrospective trial registration, which may affect the prespecification of outcomes and analysis plans, although all study procedures were predefined. Another limitation of this study is the presence of a baseline difference in buccal bone height between groups, which may affect comparability; although percentage change analysis was performed, it does not fully account for this imbalance.

## Conclusion

Vestibular socket therapy with a pericardium membrane demonstrated promising clinical and esthetic outcomes in type II sockets; however, further studies with larger sample sizes and longer follow-up periods are recommended.”

## Data Availability

The dataset used and/or analyzed in the current study are available from the corresponding author upon request.

## Abbreviations

VST: Vestibular socket therapy
GBR: Guided bone regeneration
PES: Pink esthetic score
mSBI: Modified sulcus bleeding index
CBCTZ: Cone Beam Computed Tomography
SD: Standard deviations
IQR: Interquartile ranges
